# Mechanisms of Generating Polyubiquitin Chains of Different Topology

**DOI:** 10.3390/cells3030674

**Published:** 2014-07-01

**Authors:** Randy Suryadinata, Siti Nur Ain Roesley, George Yang, Boris Šarčević

**Affiliations:** 1 CSIRO Materials Science and Engineering, Parkville, Victoria 3052, Australia; 2 Cell Cycle and Cancer Unit, St. Vincent’s Institute of Medical Research, Fitzroy, Victoria 3065, Australia; 3 Department of Medicine, St. Vincent’s Hospital, University of Melbourne, Fitzroy, Victoria 3065, Australia

**Keywords:** protein ubiquitination, ubiquitin chain topologies, polyubiquitination

## Abstract

Ubiquitination is an important post-translational process involving attachment of the ubiquitin molecule to lysine residue/s on a substrate protein or on another ubiquitin molecule, leading to the formation of protein mono-, multi- or polyubiquitination. Protein ubiquitination requires a cascade of three enzymes, where the interplay between different ubiquitin-conjugating and ubiquitin-ligase enzymes generates diverse ubiquitinated proteins topologies. Structurally diverse ubiquitin conjugates are recognized by specific proteins with ubiquitin-binding domains (UBDs) to target the substrate proteins of different pathways. The mechanism/s for generating the different ubiquitinated proteins topologies is not well understood. Here, we will discuss our current understanding of the mechanisms underpinning the generation of mono- or polyubiquitinated substrates. In addition, we will discuss how linkage-specific polyubiquitin chains through lysines-11, -48 or -63 are formed to target proteins to different fates by binding specific UBD proteins.

## 1. Introduction

Ubiquitination is a fundamental post-translational modification which controls nearly all vital cellular processes, such as cell survival, signaling and the cell cycle [1]. Protein ubiquitination requires a cascade of three classes of enzymes [1]. First, the 8 kDa ubiquitin (Ub) forms a thioester bond with the catalytic cysteine of an E1 ubiquitin-activating enzyme in an Adenosine-5’-triphosphate (ATP)-dependent manner. The Ub is then transferred from the E1 to the active site cysteine of the E2 ubiquitin-conjugating enzyme through a transesterification reaction. Finally, the substrate-binding E3 ligase cooperates with E2s to facilitate the transfer of Ub onto a substrate lysine (K) to form an isopeptide bond, resulting in protein ubiquitination. There are three major E3 ligase families. The RING (really interesting new gene) E3 ubiquitin-ligase, which lacks the catalytic cysteine, recruits both the E2~Ub conjugate and protein substrate to mediate the formation of an isopeptide bond between the C-terminal glycine of Ub and the -amino group of a lysine residue on the substrate. In contrast, HECT (homologous to E6-AP carboxyl terminus) E3s first accept Ub from the E2~Ub conjugate onto a catalytic cysteine via transesterification reaction, and then transfer the Ub to a substrate lysine [1]. More recently, hybrid RING/HECT E3 ligases, referred to as the ring-between-ring (RBR) E3 ligases have been characterized [2]. Typically, RBR E3 ligases contain two RING domains separated by the so-called in-between ring (IBR) domain [2,3]. The N-terminal RING1 domain adopts the functionality of the canonical RING E3 ligases for binding and recognizing the E2~Ub conjugate, while the RING2 domain possesses a catalytic cysteine for accepting a Ub molecule from the E2 before transferring onto the substrate lysine [2,3].

## 2. Structural Versatility of Protein Ubiquitination

**Figure 1:**
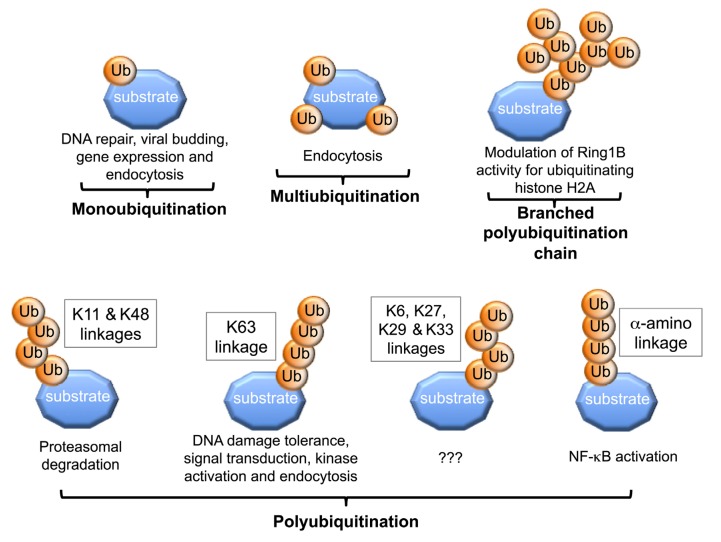
Generation of different Ub structures leads to different protein fates.

Protein ubiquitination is a versatile process due to the ability of the Ub molecule to be conjugated onto substrate lysine/s, or onto itself through its own lysines or via its N-terminal methionine residue, to generate a diverse range of structures [4]. This in turn regulates the fates of the protein substrates by targeting them to different signaling pathways. For example, attachment of a single or several Ub/s onto substrate lysine/s results in monoubiquitination or multiubiquitination, respectively [4,5,6]. These modifications can regulate cellular processes such as DNA repair, regulation of histone function, gene expression and receptor endocytosis [7,8,9,10]. In contrast, polyubiquitination occurs when the lysines on the substrate-conjugated Ub act as acceptors for sequential rounds of catalysis resulting in the formation of poly-Ub chains [1,4,6]. An acceptor Ub refers to a Ub molecule in which the ε-amino group of the lysine or the α-amino group of the N-terminal methionine forms an isopeptide bond with the C-terminal glycine of the donor Ub. Formation of the linear head-to-tail poly-Ub chains, where the C-terminal glycine of Ub is attached to the α-amino group of the N-terminal methionine residue of another Ub, can be catalyzed by the RBR E3 ligase HOIL/HOIP/SHARPIN linear Ub chain assembly E3 ligase complex (LUBAC). This is important for activation of the NF-κB transcription factor [11,12,13,14,15]. Poly-Ub chains of different structural topology can be generated, since any one of the seven lysines on Ub, positioned at amino acid residues 6, 11, 27, 29, 33, 48 and 63, may be utilized [16]. Different chains types are in turn recognized by different UBD proteins, targeting the substrate protein to specific signaling pathways. For example, polyubiquitination through Ub K48, with a chain of at least 4 Ub molecules, generally targets proteins for proteasomal degradation [17]. Similarly, K11-linked polyubiquitination of proteins can also result in their proteasomal degradation [18]. On the other hand, K63-linked poly-Ub chains lead to non-degradative cellular processes, including kinase activation, DNA damage tolerance, signal transduction and endocytosis [7]. In addition, branched Ub chains can also be generated during polyubiquitination. Indeed, auto-ubiquitination of the RING E3 Ring1b generates branched Ub chains via Ub lysines-6, -27 and -48, which is crucial for its ligase activity to monoubiquitinate histone H2A [19] (Figure 1). Recent studies have also shown that the anaphase-promoting complex (APC) E3 ligase can generate branched Ub chains, which enhances their recognition and degradation by the proteasome compared to homogeneous chains linked through K11 [20].

Attachment of single or multiple Ub/s onto substrate lysine/s (K) results in mono- or multi-ubiquitination, leading to diverse cellular responses, including DNA repair, viral budding, gene expression and endocytosis. Further attachment of Ub molecules to each other leads to polyubiquitination. Formation of poly-Ub chains via Ub K11 or K48 results in proteasomal degradation, while K63-linked poly-Ub chains can regulate DNA damage response signaling. Linear N-terminus head-to-tail poly-Ub chains regulate NF-κB activation. Branched polyubiquitination via K6, K27 and K48 occurs on RING E3 Ring 1b ligase, which is required to modulate its activity in monoubiquitinating histone 2A.

## 3. Molecular and Structural Insights into Protein Ubiquitination

While the importance of ubiquitinated protein structural diversity in controlling the specific fates of target proteins is clear, the mechanisms which underpin the generation of these different structures is not fully understood. In mammals, there are over 30 E2s and over 500 E3s [21,22,23,24,25,26,27], and the combinatorial effect of the different E2s and E3s is responsible for generation of different ubiquitinated protein structures. For example, some E2/E3 pairs are only able to catalyze the initial Ub attachment onto the substrate. Indeed, the E2 Rad6, in association with the RING E3 Rad18, promotes monoubiquitination of the proliferating cell nuclear antigen (PCNA) on lysine-164 in response to replication fork stalling or DNA damage [28,29,30]. Despite being intrinsically capable of catalyzing Ub chain formation, the E2 Rad6 is biased towards monoubiquitination due to the inhibitory effect induced by Rad18 binding, which competes with acceptor ubiquitin for a non-covalent “backside” binding site on Rad6 [31]. Monoubiquitinated PCNA is subsequently recognized by the E2 Ubc13/Mms2 complex, which together with its RING E3 ligase Rad5, promote K63-linked poly-Ub chain formation [28]. There are also examples where different E2s, upon association with the same RING E3 ligases, dictate the different stages of polyubiquitination. For example, the human SCF^βTrCP2^ RING E3 ligase initially recruits the E2 Ubc5 to catalyze the attachment of Ub onto lysines-21/22 of IκBα. The SCF^βTrCP2^ then recruits Cdc34 to catalyze the formation of K48-linked poly-Ub chain [32]. Similarly, the yeast APC E3 ligase initially recruits the E2 Ubc4 to catalyze monoubiquitination of mitotic substrates such as securin and cyclin B, and subsequently recruits Ubc1 to promote polyubiquitination via Ub K48 [33].

In addition to the aforementioned combinatorial E2/E3 pairs which are biased towards either mono- or -polyubiquitinating activities, certain E2/E3 pairs demonstrate dual-functionality and are capable of catalyzing substrate ubiquitination and poly-Ub chain extension. Hence, the yeast E2 Cdc34, together with its cognate Skp-cullin-F-box^Cdc4^ (SCF^Cdc4^) RING E3 ligase, is capable of catalyzing the ubiquitination of lysines on its substrate Sic1 as well as the subsequent formation of the K48-linked poly-Ub chains [34,35,36]. Studies have revealed that the structure of the catalytic core of Cdc34 plays a critical role in determining the selection of lysines on substrate protein and lysine-48 on Ub. As such, mutation of a single residue in Cdc34 catalytic site can dictate the catalytic activity of this enzyme as either a monoubiquitinating or polyubiquitinating enzyme [36,37].

### 3.1. Lysine Selection during Protein Mono- and Multi-Ubiquitination

Although the precise mechanisms utilized by the different repertoire of E2s and E3s to either mono- or poly-ubiquitinate proteins with different chain topologies are not fully defined, emerging molecular and structural studies have provided mechanistic insights into protein ubiquitination.

The attachment of Ub onto specific substrate lysines is dependent on determinants within E2s, E3s and the substrate. Certain E3 ligases play a critical role in substrate selection and the positioning of specific lysine/s for ubiquitination. For example, the F-box substrate-binding subunits of the SCF RING E3s family are responsible for optimally positioning one or more substrate lysine/s for ubiquitination by the bound E2~Ub conjugate. Indeed, ubiquitination of β-catenin and IκBα on specific lysines is mediated by their binding to a site on the F box βTrCP subunit, which positions a specific ubiquitinated lysine toward the E2~Ub thioester bond [38]. Conversely, the F-box protein Cdc4 contains several binding sites for its substrate, the cyclin-dependent kinase inhibitor Sic1, allowing multiple binding geometries and thus ubiquitination of numerous Sic1 lysine residues [39]. The attachment of Ub on multiple substrate lysines can also occur through dimerization of the RING E3/E2 complexes [39,40]. In addition to structural determinants within the E3 ligase, determinants within the substrate are equally important for regulating lysine selection. Therefore, residues proximal to lysines are critical determinants of their ubiquitination efficiency [41].

In HECT E3 ligases, lysine selectivity during ubiquitination can be mediated by structural constraints through interaction with Ub molecule as well as non-covalent inter-domain interactions, as evident in recent studies on HECT E3 Rsp5 [42,43,44]. The initial charging of the catalytic cysteine of HECT E3 Rsp5 with Ub requires two non-covalent interactions. These include binding of Rsp5 N-lobe with the E2 and interaction of the C-lobe with the E2-linked Ub molecule, thus allowing efficient transesterification of the Ub molecule onto the catalytic cysteine on Rsp5 [44]. Once charged, the C-lobe of Rsp5 remains associated with the thioester-linked Ub, leading to a ~130° rotation towards the WW-substrate binding domain on the N-lobe [42,43]. The positioning of both the C-lobe and the thioester-linked Ub restricts the binding orientations available to the WW domain, thus limiting the positions available for a lysine to access to the thioester-linked Ub [42,43]. Since Rsp5 can generate K63 poly-Ub linkages, this conformation presumably favors Ub K63 for attack of the thioester.

Conformational restraints are also important in dictating linkage specificity of the LUBAC RBR E3 ligase. The HOIP subunit within the LUBAC is responsible for aligning the Ub molecule to allow efficient nucleophillic attack towards the E3~Ub conjugate [15]. Structural analyses revealed that the helical base, together with a zinc-finger (Zf1) domain on the C-terminus of the HOIP subunit optimally orient the acceptor Ub so that the α-amino group is 3.5 Å away from the thioester forming cysteine 885 [15].

### 3.2. Mechanisms of Polyubiquitin Chain Formation

As already noted, the generation of poly-Ub chains linked to a particular Ub lysine leads to the formation of a chain of specific structural topology which can target substrate proteins to different fates. Emerging molecular, structural and modeling studies have also provided mechanistic insights into lysine selection on Ub during polyubiquitination. Several studies to date have shown that E2 enzymes can define lysine selection on Ub and therefore linkage specificity during polyubiquitination.

**Figure 2:**
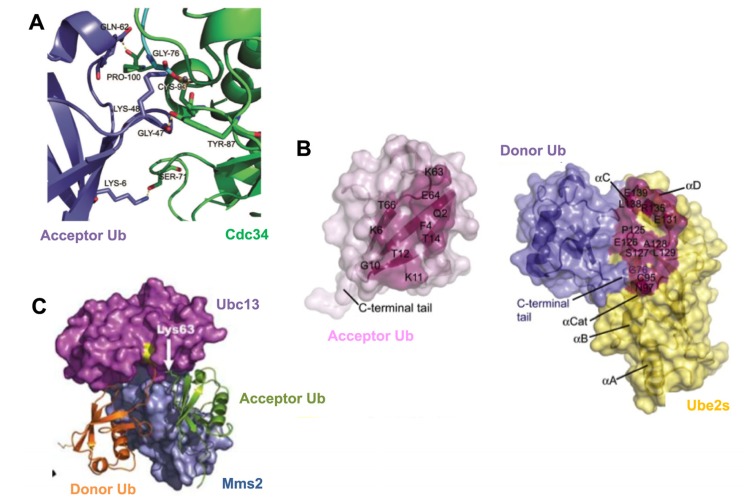
Mechanisms for generating lysine-specific poly-Ub chains.

Determinants in the catalytic core of E2s, Ub and structural features distant from the catalytic site, are important for Ub lysine specificity during polyubiquitination. For example, studies with UbcH5A, which can generate K11, -48 and -63 poly-Ub chains, demonstrate that mutation of residues near the catalytic cysteine can modulate linkage specificity of this E2 enzyme [45]. Therefore, mutation of UbcH5A serine-83, located two positions from the catalytic cysteine, to arginine, alters the preference of this enzyme towards forming K63-linked Ub chains at the expense of K11-linked Ub chains [45]. The importance of key catalytic residues in E2s for generating lysine-specific poly-Ub chains is also evident with the K48-specific polyubiquitinating enzyme Cdc34. Mutation of serine-139 in the catalytic core of yeast Cdc34 to aspartate abrogated the ability of this enzyme to generate K48-linked poly-Ub chains [36]. However, mutation of amino acids proximal to the Ub K48, including Q49P and L50S, restored the activity of this Cdc34 mutant in generating Ub K48-linked chains. Therefore, residues in the catalytic site of E2s can play critical roles in lysine selectivity on substrate or Ub. In addition to the importance of amino acids proximal to Ub K48, mutation of other Ub residues, including lysine-6 and glutamine-62, leads to a reduction in the ability of Cdc34 to generate K48-linked poly-Ub chains [36,41]. Recent molecular modeling and biochemical studies have proposed a model for the interaction of the Cdc34 catalytic site with acceptor Ub. Serine-71, tyrosine-87 and proline-100 of human Cdc34 are thought to interact with lysine-6, glycine-47 and glutamine-62 of acceptor Ub, respectively, to optimally position Ub K48 of acceptor Ub toward the Cdc34~Ub thioester bond to catalyze K48-specific Ub chain formation [41] (Figure 2A). Additionally, an acidic loop region near the Cdc34 catalytic site is also critical for generating K48-linked poly-Ub chains [34].

Modeling and biochemical studies have also been utilized to understand how the E2 Ubc1 forms K48-linked poly-Ub chains [46]. This enzyme depends on a polar cluster of residues in close proximity of the catalytic cysteine [46]. Mutation on Ubc1 threonine-84, glutamine-122 or alanine-124 significantly reduces K48-linked poly-Ub formation [46]. Ub residues are also important for K48-specific chain formation by Ubc1 [46]. For example, the aromatic side chain of tyrosine-59 on Ub is thought to provide optimal orientation for productive attack by Ubc1 through molecular interaction with Ubc1 threonine-84 [46]. Altogether, compatibility between key residues in the catalytic core of E2s and those surrounding the ubiquitinated lysine are important determinants during K48-linked polyubiquitination [37].

(**A**) Computational docking of the human E2, Cdc34 (green), in complex with an acceptor Ub (purple). Serine-71, tyrosine-87 and proline-100 on human Cdc34 interact with lysine-6, glycine-47 and glutamine-62 on acceptor Ub, respectively, to align K48 of acceptor Ub in the Cdc34 catalytic site and promote K48-linked poly-Ub chain formation [33]. (**B**) Surface representation of an acceptor Ub (pink, left) and the donor Ub (blue) in complex with Ube2s (yellow, right), based on computational docking and mutational analysis. The residues that make intermolecular contacts are highlighted, which allow optimal positioning of acceptor Ub K11 toward the Ube2S~Ub_donor_ thioester bond to catalyze K11-specific poly-Ub chains [36]. Copyright (2011), with permission from Elsevier (**C**) Crystal structure of Mms2 (blue) and Ubc13 (magenta) in association with acceptor Ub (green) and a donor Ub (orange). Acceptor Ub is bound by Mms2 and Ubc13 to position K63 toward the Ubc13~Ub_donor_ thioester bond [38]. Copyright (2006), with permission from Nature Publishing Group.

In addition to the importance of the determinants in the E2 catalytic core for aligning particular Ub lysines to generate chains of specific topology, emerging studies have also demonstrated that this specificity can also be determined by amino acids in Ub, which directly contribute to catalysis. For example, the E2 Ube2S interacts with a specific recognition sequence on acceptor Ub termed the TEK-box, to generate of K11-specific poly-Ub chains [47]. Interestingly, molecular modeling studies suggest that, in addition to K11, other lysines of the acceptor Ub can also be exposed to the Ube2S active site. However, K11-linkage specificity is still achieved due to a substrate-assisted catalysis. As such, glutamate-34 of acceptor Ub assists in the optimal positioning and pKa suppression of Ub K11 to allow its nucleophillic attack of the Ube2S~Ub thioester bond [47] (Figure 2B).

Key auxiliary subunits can also control Ub lysine linkage specificity during polyubiquitination. For example, the E2 Ubc13 generates K63-linked poly-Ub chains by forming a hetero-dimer with the Ub-conjugating enzyme variant (UEV) Mms2, which imposes structural constraints on acceptor Ub for insertion of lysine-63 into the Ubc13 active site [48,49]. Mms2 makes significant contact of 2,282 Å^2^ with the acceptor Ub, to position the K63 residue of the acceptor Ub towards the Ubc13~Ub thioester bond to ensure K63-specific Ub chain formation (Figure 2C).

## 4. The Impact of Different Chain Topology

**Figure 3:**
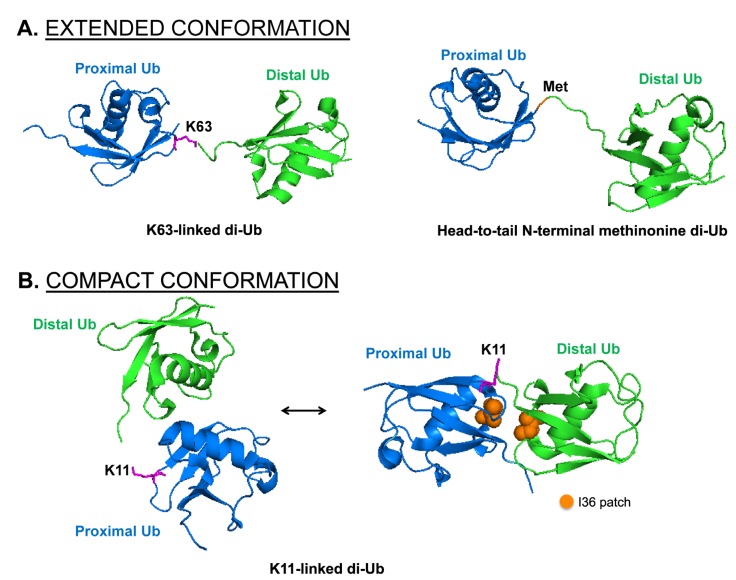
Different conformations of di-Ub structures.

Structural studies reveal that poly-Ub chains of specific linkage adopt extended or compact conformations, which mediates differential recognition by proteins with specific UBDs. K63-linked and head-to-tail N-terminal methionine poly-Ub chains adopt extended conformations allowing a high degree of structural flexibility [50,51,52]. This extended conformation relies predominantly on the isopeptide bond between the C-terminal glycine of the distal (donor) Ub and the lysine or methionine of the proximal (acceptor) Ub, with minimal contact between the two Ub molecules (Figure 3A). Conversely, K6-, K11- and K48-linked poly-Ub chains adopt a relatively compact conformation, due to additional contact points on the surface of the adjacent Ub molecules. In many cases, these unique contact points consist of hydrophobic patches on Ub. Three hydrophobic patch regions mediating these interactions have been defined on Ub. These include the phenylalanine-4 (F4) patch, comprising glutamine-2, phenylalanine-4 and threonine-12 [53]; the isoleucine-36 (I36) patch, comprising isoleucine-36, leucine-71 and leucine-73 [54]; and the isoleucine-44 (I44) patch, comprising leucine-8, isoleucine-44, histidine-68 and valine-70 [55]. Structural studies of the K11-linked di-Ub (Ub_2_) complex reveals two distinct compact conformations mediated through interaction of the α-helix interface [56], or via the I36 patch of Ub [57,58] (Figure 3B). Both conformations coexist in equilibrium, with solvent exposure of the different hydrophobic regions allowing interaction with various UBD-containing binding partners [56,57]. Similarly, K48-linked poly-Ub chains adopt a compact conformation predominantly mediated by interaction via the I44 hydrophobic patch [51,59,60]. NMR studies have revealed an additional, less preferred conformation on K48-linked di-Ub, induced by asymmetric interaction between the I36 and I44 patches of the distal and proximal Ubs [58,60,61,62]. Interestingly, recent studies demonstrated that, similar to K48-linked chains, both K63-linked and head-to-tail N-terminal poly-Ub chains exist in two conformational geometries [63].

(**A**) Crystal structures of the extended conformations adopted by K63-linked (left, PDB: 2JF5), and head-to-tail N-terminal methionine (right, PDB: 2W9N) di-Ub chain structures [39]. (**B**) The K11-linked di-Ub complex has 2 distinct compact conformations mediated by α-helix interactions (left, PDB: 2XEW) [45] or through interaction of the I36 hydrophobic surface patch (orange spheres) on proximal (blue) and distal (green) Ubs (right, PDB: 3NOB) [46].

### 4.1. Recognition Pattern for Different UBD-Containing Proteins

**Figure 4:**
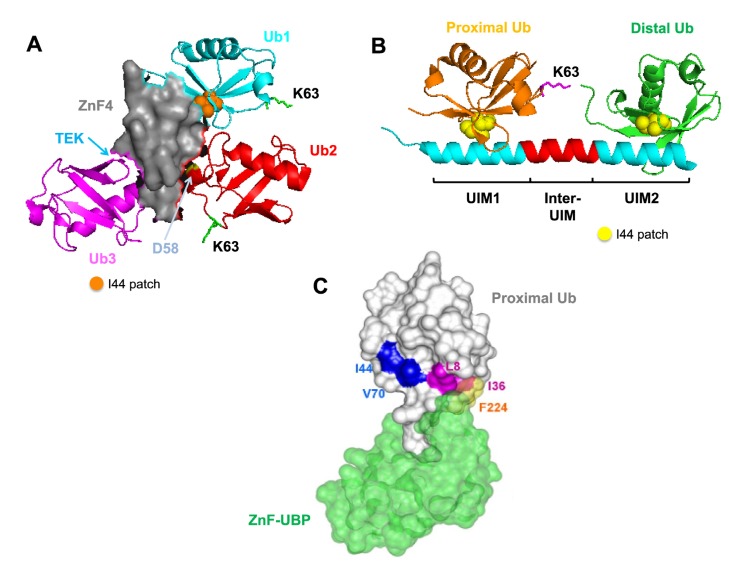
Recognition of specific Ub chains by UBD-containing proteins.

Proteins containing diverse UBDs specifically bind to poly-Ub chains of different structural topology to target polyubiquitinated proteins to different signaling pathways and fates. To date, over 200 proteins with at least 20 different types of UBDs have been identified, which bind to different Ub structures in a non-covalent manner [64]. UBD-containing proteins recognize structural determinants and unique local features within Ub chains to differentiate chains of different linkage type and structural topology. For example, a single Zinc finger 4 (ZnF4) domain of the Ub editing protein A20 uniquely recognizes K63-linked chains through interaction with the I44 patch, an aspartate-58 and the TEK box from each individual Ub molecule [50,65] (Figure 4A). The unique surface determinants are also important for recognition and chain selectivity by the Ub-pathway associated (UBA) domains [66,67]. Therefore, the exposed I44 patch of the proximal Ub, and valine-70 and leucine-73 residues of the distal Ub allow binding and positioning of the UBA2 domain of the proteasomal signaling protein hHR23A between the K48-linked chains [67]. This so-called ‘sandwich-like’ conformation is also evident in binding of the K48-linked di-Ub by the UBA domain on *S. pombe* Mud1 [66]. The requirement for unique features for poly-Ub chain recognition is also exemplified by the Ub binding of the ABIN and NEMO (UBAN) motifs for recognition of linear head-to-tail poly-Ub chains. Two UBAN motifs are aligned through homodimerization of two NF- κ B essential modulator (NEMO) proteins, where one UBAN motif recognizes the I44 hydrophobic patch of the distal Ub and the other motif binds F4 patch of the proximal Ub [68]. Despite being similar in their overall extended chain structure and topology, K63-linked chains exhibit weak interaction with the UBAN-containing NEMO homodimer, due to the unavailability of F4 hydrophobic patch on the surface of the proximal Ub [50,69].

(**A**) A20 ZnF4 domain (grey) recognizes K63-linked triubiquitin (PDB: 3OJ3). This occurs through the interaction of the I44 hydrophobic patch (orange sphere) of Ub_1_ (cyan), aspartate-58 on the 50s loop (yellow) of Ub_2_ (red) and the TEK-box of Ub_3_ (magenta). The positions of the putative K63 linkages are indicated [53]. (**B**) Crystal structure of the tandem UIMs (cyan) on Rap80 protein in complex with the extended K63-linked di-Ub chain (PDB: 3A1Q). Two UIM domains are separated by a 7 residue helix (red) for optimal recognition of the I44 patch (yellow spheres) on the proximal (orange) and distal (green) Ubs [58]. (**C**) ZnF-UBP (green) from the deubiquitinating enzyme IsoT recognizes and binds to the C-terminal glycine of a proximal Ub (grey) (PDB: 2G45). This occurs through the interaction of L8 and I36 of the Ub (magenta) and F224 of the ZnF-UBP (yellow) [64]. Copyright (2006), with permission from Elsevier.

As discussed, Ub chains can adopt extended or compact structural conformations. The distance between two Ub molecules on a chain is also an important determinant in defining UBD binding specificity. Some proteins may contain a defined spacer separating two UBDs to allow optimal recognition and binding to a specific poly-Ub chain [70,71]. These UBDs, also referred to as the Ub-interacting motif (UIM), generally exist as a folded α-helix with a hydrophobic binding region, which can recognize the I44 patch on Ub [72]. For example, the tandem UIMs on Rap80 protein are separated by a seven-residue helix for optimal recognition of the extended K63-linked, but not the compact K48-linked chains [70,71] (Figure 4B). In contrast, the two UIMs on the ataxin-3 protein are separated by only two residues, thus allowing specific recognition of the compact K48-linked Ub chains [71].

Poly-Ub chain conformational flexibility can also be an important determinant for recognition by different UBD-containing proteins. For example, the single Npl4-like zinc finger (NZF) domain on TAB2 and TAB3 adaptor proteins can distinguish the structurally similar K63-linked and linear head-to-tail (N-terminal methionine) poly-Ub chains [73]. Structural flexibility of the K63-linked chains allows perpendicular “bending” to promote interaction between the exposed I44 patch on di-Ub with surface determinants on a single NZF domain [74,75]. Conversely, such structural flexibility is not possible for the linear head-to-tail poly-Ub chains, since this would displace the methionine of the proximal Ub from the distal Ub [74,75].

UBD-containing proteins are also able to interact with unanchored Ub chains. This is illustrated by the zinc finger ubiquitin binding protein (ZnF-UBP), from the deubiquitinating enzyme isopeptidase T (IsoT), which recognizes and binds to the free C-terminal glycine region of the proximal Ub at nanomolar affinity [76,77] (Figure 4C). This promotes the disassembly of the unanchored poly-Ub chain initiated from the proximal unattached end [76,77].

## 5. Conclusions

Protein ubiquitination is a versatile post-translational modification and the mechanisms underpinning the generation of structurally diverse protein-Ub structures are slowly emerging. This versatility is mediated by combination of numerous E2 Ub-conjugating and E3 Ub-ligase enzymes, and in some cases accessory proteins. Although biochemical, structural and genetic studies have provided significant insights into mechanisms of protein ubiquitination and how these diverse modifications bind specific proteins to regulate different cellular pathways, significant issues are yet to be resolved. For example, what are the key enzymes that generate K6-, K27-, K29- and K33-specific poly-Ub chains and what are their major biological roles in addition to their purported role in proteasomal degradation [78]? Some insights have also been provided into the role of branched poly-Ub chains; however, the mechanism for generating these structures is unclear. Further detailed structural information on different ubiquitination enzymes and how they associate with substrates and Ub will be required to gain a full insight into protein-Ub structural diversity. This knowledge will be important for increasing our understanding of the role of ubiquitination in normal cellular processes and how perturbation of this pathway is linked to the development of pathologies, such as cancer, inflammatory and autoimmune diseases. The successful clinical use of the proteasomal inhibitor bortezomib for the treatment of multiple myeloma [79] and emerging studies suggesting that enzymes in the ubiquitination pathway may be viable therapeutic targets augurs well for the development of new compounds to treat different pathologies.
